# Ravinder Lal Kapur, M.D. 1938-2006

**Published:** 2007

**Authors:** Ajit V Bhide

**Affiliations:** E-mail: mukunjit@yahoo.com

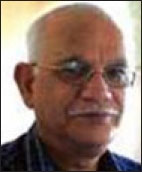



Prof. Ravinder Lal Kapur expired on Friday, 24 November 2006, at Bellagio, Italy, while on a sabbatical. The rude shock of this sudden demise from myocardial infarction shook his immediate family and a huge fraternity of friends, colleagues, students, patients and admirers. Our hearts go out to his beloved wife, Prof. Malavika Kapur, a clinical psychologist, Dr. Svapna Sabnis, paediatrician, his daughter and Dr. Sharad Kapur, his IT entrepreneur son.

The fourth of five children and the only son of a respected doctor, he was born in pre-partition West Punjab in 1938. For many years later, Ravi would recall the horrors of the partition he saw at very close quarters, when he had to flee to India on a train. It shaped his attitude to violence, which he abhorred with an uncommon passion. He later sublimated this sense of horror by working with terrorists, trying to understand their psyche.

The family eventually settled in Amritsar, where his father's practice picked up. Ravi did his medical graduation there and excelled in studies as well as in extra curricular activities, particularly singing and dramatics.

After his medical degree, he was determined to take up psychiatry despite many of his clinical professors urging him to take up their respective specialities.

Being preoccupied with the need to understand the mysteries of the mind, the advice of Prof. Vidyasagar and Prof. Neki spurred him to seek admission at the distant All India Institute of Mental Health (the present NIMHANS, already a Mecca for psychiatry and clinical psychology), at Bangalore. At AIIMH, he not only won a distinction at the D. P. M exam, but also the heart of Malavika Karanth, a clinical psychology student whom he married.

## Baroda, Edinburgh, Manipal and NIMHANS

After a short stint at the Sayajirao Medical College at Baroda, he earned a Commonwealth Scholarship for higher studies, the first psychiatrist to do so, and this took him to Edinburgh where he got his PhD, under Prof. Norman Kreitman. Just before his thesis defence, he suddenly took ill and was laid up in bed. In an unusual gesture, his examiners came to his bedside to have him defend the thesis, as his work had been meticulous and they did not want to postpone the exam!

Returning to India after five years, as Field Director of an Edinburgh-Manipal Psychiatric Research Project, he embarked on a major psychiatric epidemiological study. Upon completion of the project, he was recruited at the Kasturba Medical College, Manipal, as a professor of psychiatry, heading the department there for four years.

He then joined NIMHANS as professor of Community Psychiatry in 1976.

Epidemiological work carried out in the area of Kota in the South Kanara district during his days at Manipal, led to the publication of the first Indian monograph on psychiatric epidemiology, *“The Great Universe of Kota”* (with Prof. G. M. Carstairs, Hogarth Press, 1976). This milestone book created a stir because of some unusual ideas. The medical establishment had always held traditional healers in disdain. The “Kota” book advocated integrating them in mental health care. This work also set in motion the Indian Psychiatric Survey Schedule, the first instrument for such surveys devised entirely indigenously, by Prof. Kapur and his team.

The next year, he became head of the Department of Psychiatry at NIMHANS and was till then the youngest person to ever head a postgraduate department of psychiatry in the country. He continued at NIMHANS till 1983.

A witty and erudite speaker, he was swift in grasping concepts and conveying them in simple elegant language. He was intolerant of the bombastic and the pompous. During his days as professor at NIMHANS, an atmosphere was created mainly by him, that encouraged intellectual debate, gave rise to phenomenal teaching programmes that have stood the test of time and everyone from the junior most students to the senior most faculty enjoyed great freedom of expression.

Many revolutionary changes were on at NIMHANS in his time and his keen interest in psychosocial aspects of psychiatry was wrongly interpreted as being in opposition to the swelling tide of biological psychiatry. He was, in fact, instrumental in setting up the department of psychopharmacology at NIMHANS, which is now very well known. He weathered many storms in those days, never relinquishing his stance supporting a multidisciplinary approach and minimising role hierarchy.

## Awe, Friendliness and Ethical Practice

All those who worked closely with him remember the awe the man generated at first encounter; this soon gave way to disarming friendliness that was his hallmark. Never before or after, have I seen a ‘no-nonsense’ attitude combined with alacrity and informality all at once. Many of his students, who are now heads of units and departments, acknowledge his being their role model. As a thesis guide, examiner and chairman of academic sessions, he was known to be stimulating though unsparing in his criticism, which never failed to be constructive. He was always greatly delighted with his students’ achievements but lamented at times that some did not keep in touch with him. Dr. Kapur espoused the cause of ethical practice with passion. Intellectual dishonesty irked him no end and would cast him into very brief spells of cynicism.

He served on the editorial board and board of reviewers of many publications (including the *Mens Sana Monographs*) and was one of the founding editors of ‘*Culture, Medicine and Psychiatry*’.

After NIMHANS, for a year, he was a faculty member at the Centre for Theoretical Studies at the Indian Institute of Science (IISc), Bangalore. This was also the time he entered private practice, which he carried on till the very end.

He joined the National Institute of Advanced Studies (NIAS), at the IISc campus in Bangalore, when it was founded in 1988 and was later its Deputy Director. At the time of his passing away, he was Emeritus Professor there. In this institute he could pursue his polymathic interests and interact with bright minds from varied disciplines. He was highly popular as a great synthesizer of knowledge among the faculty there and much sought after for his innovative ideas.

Prof. Kapur had also held visiting professorships at various institutions including Harvard University and had been a specialist consultant to the WHO.

## Epidemiology, Research Methodology, Yoga, Terrorism and Spirituality

Epidemiology and research methodology fascinated him for all his professional life and he had a keen interest in cross-cultural psychology and psychiatry. He often expressed alarm that psychotherapy was not given enough attention both in training and practice. An ardent practitioner and student of yoga, he was the first and possibly the only psychiatrist to have taken a sabbatical, under the aegis of ICMR, to study the subjective experiences of yogic practices. Some experiences after yoga were very alarming to him and he would call me with great urgency to share these. Those were the days before pagers and mobiles and he would grow tense and impatient if I was delayed in reaching him. We'd commiserate about these and he would not rest till he had found some explanation and/or recorded his experience.

He continued to choose unusual areas to explore, especially after his NIMHANS days. Among these was work trying to understand the psyche of terrorists in Punjab (and in the context of this work, the study of the alienation of youth in late twentieth century India), studies of senior administrative officers’ job orientation and satisfaction from a psychological perspective, psychological scrutiny of creativity among Indian scientists and spirituality research. He interviewed the Dalai Lama with great savvy for an international news channel and later held a no holds barred interview with the redoubtable K. P. S. Gill who was instrumental in containing terrorism in Punjab, in the early 1990s. All these pursuits sometimes earned him the sobriquet of being a ‘fringe psychiatrist’; an unfair sobriquet, when one recalls that he had never forsaken hard-core psychiatry, was always happy to be back in his clinical practice and relished clinical discussions.

For well over a decade, he had been pursuing his interest in studying spirituality and was researching the lives of *rishis* and *sadhus* mainly in the Himalayas, expanding on the mental health implications of his findings. He undertook several Himalayan sojourns to catch up on his subjects and even in his sixties, seemed youthful and rejuvenated after each mountain trip. His interest in spirituality never distanced him from his abiding faith in rationality. This spirit of faith without superstition was instilled in him by his parents, particularly his mother. At Bellagio, he was actually, on assignment, writing about his research with *rishis* and *sadhus*. A book he planned to publish on his work, was to be titled “*Living another way: The Life of a Sanyasin*”. Less than a month earlier, he had delivered a memorable lecture on this very topic, at the Annual Conference of the IPS-South Zone, at Mangalore.

## Music, Books and Sri Aurobindo

Dr. Ravi Kapur was a great lover of music and he was learning Hindustani vocal for several years. He sang rare numbers and relished the voices of Saigal, Reshma, Asha Bhonsle, Manna Dey and Pankaj Malik. He enjoyed listening to Marathi *Natya Sangeet* thoroughly, though he didn't really follow the language. I remember about seven years ago when we were staying at the same hotel for a conference, I heard the sonorous lyrics, “*Pyaar to hona hi thaa…*” belted out loud and tuneful, rising from the well of the stairs. Sure it was some young student, I ran to catch the owner of the voice and was stunned to see Ravi prancing up the steps totally absorbed in his singing.

He and his wife loved entertaining people at “Balavana”, their home. A gracious host and remarkable conversationalist, he had the ability to ‘walk with kings, not lose the common touch’. Despite having walked with umpteen kings, he never dropped names. He did drop into our place, often at short notice and spiritedly partook of our meal. Full of ideas and anecdotes, he was always thirsting for lively interaction.

He would often recall with gratitude the values instilled in him by his parents: rationality and discipline from his father and a spiritual perseverance from his mother. Dr. Kapur used to list Sri Aurobindo, the late Prof. N. C. Surya and Prof. J. S. Neki as important influences in his life. He told me that he had learnt a great deal also from Prof Neki's poetry in his practice of healing and his outlook to life.

A romantic at heart, Ravi savoured the poetry of Harivanshrai Bachchan, Sahir Ludhianvi, Kaifi Azmi and Faiz Ahmed Faiz; the latter, he would quote aptly and promptly on many occasions. He was also enamoured of the poetry of his friend Ronald Laing, though he disapproved of the latter's disavowal of psychiatry.

He loved his books and appreciated particularly C. P. Snow's “*The Two Cultures and the Scientific revolution*”. He cherished its main premise that the chasm between the sciences and the humanities needed to be logically bridged. To every new student of psychiatry, Ravi's impassioned message was that psychiatry was both a science and an art. In the less serious realm, he was a fan of Spike Milligan, Peter Sellers and P. G. Wodehouse.

## Awards and Conference Organisation

The very first recipient of the Marfatia Award (1971), he also received the Bhagwat Award (1983) and the late Dr. D. L. N. Murthy Rao Memorial Oration Award (1994), of the Indian Psychiatric Society. The Karnataka Chapter of IPS had bestowed on him its Eminent Psychiatrist Award (1993) and the Dr. Achar Memorial Oration Award (1998).

He was the Chairman of the Annual Conference of the Indian Psychiatric Society Organizing Committee in 1996. As Organizing Secretary, I cherished my association with him. He joined me in paying attention to every detail of organising what was, till then, the biggest national conference of psychiatrists. I remember his buoyant voice when he called excitedly from Delhi to inform me that the then Vice-President of India, Dr. K. R. Narayanan, had agreed to inaugurate the conference! He organized at NIAS, the first ever international conference on qualitative methods in mental health research in 1998.

## In Closing

Prof. Kapur died, ‘a soldier,’ as our senior member Col D. S. Goel noted, ‘with his boots on’. Col Goel has also aptly called Ravi Kapur, ‘the Bertrand Russell of Indian psychiatry’.

Personally, I have lost a mentor, a dear friend and the most dependable sounding board; and my family has lost a father figure. The greatest lesson I learnt from Ravi is not to get immobilised, for life must move on.

Farewell to thee, Master. We thank Providence that you were ours for this while.

## About the Author

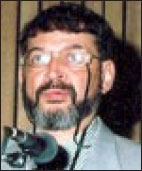



Dr. Ajit V. Bhide, heads the departments of psychiatry and family medicine at St. Martha's Hospital, Bangalore. An alumnus of St. John's Medical College and of NIMHANS, Bangalore, he has served on the faculty of both these institutions. Dr. Bhide's main areas of interest are child and adolescent mental health, family issues in psychiatry, psychotherapy, preventive medicine and media and mental health. He is also deeply interested in literature, history, the performing arts and evolutionary biology.

